# Red meat, poultry and fish consumption and risk of diabetes: a 9 year prospective cohort study of the China Kadoorie Biobank

**DOI:** 10.1007/s00125-020-05091-x

**Published:** 2020-01-22

**Authors:** Huaidong Du, Yu Guo, Derrick A. Bennett, Fiona Bragg, Zheng Bian, Mahmuda Chadni, Canqing Yu, Yiping Chen, Yunlong Tan, Iona Y. Millwood, Wei Gan, Ling Yang, Pang Yao, Guojin Luo, Jianguo Li, Yulu Qin, Jun Lv, Xu Lin, Tim Key, Junshi Chen, Robert Clarke, Liming Li, Zhengming Chen, on behalf of the China Kadoorie Biobank collaborative group

**Affiliations:** 1 Medical Research Council Population Health Research Unit at the University of Oxford, Oxford, UK; 2 Clinical Trial Service Unit and Epidemiological Studies Unit (CTSU), Nuffield Department of Population Health, University of Oxford, Old Road Campus, Oxford, OX3 7LF UK; 3 Chinese Academy of Medical Sciences, no. 9 Dong Dan San Tiao, Dong Cheng District, Beijing, 100730 China; 4 Department of Epidemiology and Biostatistics, Peking University Health Science Center, Peking University, Beijing, China; 5 Wellcome Trust Centre for Human Genetics, University of Oxford, Oxford, UK; 6 Pengzhou CDC, Chengdu, Sichuan Province China; 7 NCDs Prevention and Control Department, Liuzhou CDC, Guangxi Autonomous Zone China; 8 CAS Key Laboratory of Nutrition, Metabolism and Food Safety, Shanghai Institute of Nutrition and Health, Chinese Academy of Sciences, Shanghai, China; 9 Cancer Epidemiology Unit, Nuffield Department of Population Health, University of Oxford, Oxford, UK; 10 China National Center for Food Safety Risk Assessment, Beijing, China

**Keywords:** Biobank, China, Diabetes, Fish, Poultry, Prospective cohort study, Red meat

## Abstract

**Aims/hypothesis:**

Previous evidence linking red meat consumption with diabetes risk mainly came from western countries, with little evidence from China, where patterns of meat consumption are different. Moreover, global evidence remains inconclusive about the associations of poultry and fish consumption with diabetes. Therefore we investigated the associations of red meat, poultry and fish intake with incidence of diabetes in a Chinese population.

**Methods:**

The prospective China Kadoorie Biobank recruited ~512,000 adults (59% women, mean age 51 years) from ten rural and urban areas across China in 2004–2008. At the baseline survey, a validated interviewer-administered laptop-based questionnaire was used to collect information on the consumption frequency of major food groups including red meat, poultry, fish, fresh fruit and several others. During ~9 years of follow-up, 14,931 incidences of new-onset diabetes were recorded among 461,036 participants who had no prior diabetes, cardiovascular diseases or cancer at baseline. Cox regression analyses were performed to calculate adjusted HRs for incident diabetes associated with red meat, poultry and fish intake.

**Results:**

At baseline, 47.0%, 1.3% and 8.9% of participants reported a regular consumption (i.e. ≥4 days/week) of red meat, poultry and fish, respectively. After adjusting for adiposity and other potential confounders, each 50 g/day increase in red meat and fish intake was associated with 11% (HR 1.11 [95% CI 1.04, 1.20]) and 6% (HR 1.06 [95% CI 1.00, 1.13]) higher risk of incident diabetes, respectively. For both, the associations were more pronounced among men and women from urban areas, with an HR (95% CI) of 1.42 (1.15, 1.74) and 1.18 (1.03, 1.36), respectively, per 50 g/day red meat intake and 1.15 (1.02, 1.30) and 1.11 (1.01, 1.23), respectively, per 50 g/day fish intake. There was no significant association between diabetes and poultry intake, either overall (HR 0.96 [95% CI 0.83, 1.12] per 50 g/day intake) or in specific population subgroups.

**Conclusions/interpretation:**

In Chinese adults, both red meat and fish, but not poultry, intake were positively associated with diabetes risk, particularly among urban participants. Our findings add new evidence linking red meat and fish intake with cardiometabolic diseases.

**Data availability:**

Details of how to access the China Kadoorie Biobank data and rules of China Kadoorie Biobank data release are available from www.ckbiobank.org/site/Data+Access.

**Electronic supplementary material:**

The online version of this article (10.1007/s00125-020-05091-x) contains peer-reviewed but unedited supplementary material, which is available to authorised users.

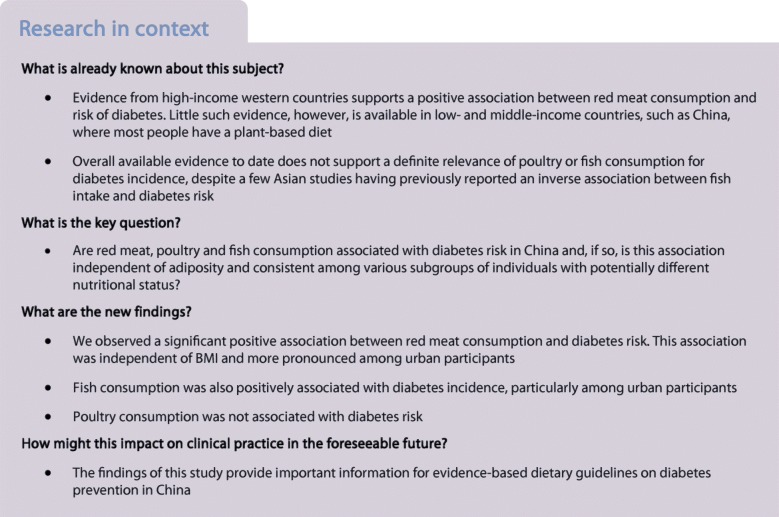



## Introduction

Diabetes is a major public health problem affecting almost half a billion people worldwide [1]. In China, the incidence of diabetes has increased rapidly since the early 1980s, currently affecting ~10% of adults [2]. About half of this increase can be attributed to increasing adiposity [3] and other lifestyle factors (e.g. smoking [4], reduced physical activity and certain dietary habits [5]), which may influence diabetes risk either independently or through adiposity.

Several prospective studies, conducted chiefly in western populations, have reported that higher consumption of red meat is associated with a higher risk of diabetes [6, 7]. Potential underlying mechanisms may include obesity and iron overload, which may lead to pancreatic beta cell dysfunction and impaired insulin sensitivity [8, 9]. In addition, other components of red meat, such as cholesterol, saturated and *trans* fatty acids, and amino acids, may also influence diabetes risk directly or through impacts on gut microbiota [6, 10]. Thus far, the only reported prospective study from China observed no such positive association [11]. People in China tend to have a lower mean BMI and consume mainly plant-based diets with lower amounts of red meat compared with people in western countries [12]. Moreover, Chinese people tend to consume pork, which contains lower amounts of iron than beef and lamb, which are more commonly consumed in western countries [13]. Therefore, reliable evidence about the association between red meat intake and risk of diabetes is particularly needed in China and other populations with relatively low body iron status [14].

The lower fat content of poultry and fish when compared with red meat has led to them being considered relatively healthier for cardiovascular disease [15, 16]. However, existing evidence does not support a clear overall association of either poultry or fish intake with diabetes risk [6, 17, 18]. We therefore investigated the associations of red meat, poultry and fish consumption with incidence of diabetes in the prospective China Kadoorie Biobank (CKB) study. In addition to the overall associations, we also examined the role of adiposity and whether sociodemographic and lifestyle factors, which are potentially related to nutritional status, might modify the associations [19].

## Methods

### Study population

The CKB is a prospective cohort study of over 0.5 million adults recruited from ten diverse areas (five rural and five urban) in China, selected to cover a wide range of risk exposures, disease patterns and stages of economic development. Details of the study design, methods and population have been previously reported [20]. In brief, between June 2004 and July 2008, all permanent residents (aged 35–74 years; not severely disabled) in pre-selected communities or villages were invited to participate in the study. Among them, about one in three (33% in rural and 27% in urban areas) responded. A total of 512,713 participants (including a few who were just outside the target age range) were included in our baseline database. All participants provided written informed consent. Regional, national and international ethics approval was obtained prior to the start of recruitment.

For the current study, we excluded participants with baseline prevalent diabetes (*n* = 30,299), ischaemic heart disease (IHD; *n* = 15,472), stroke or transient ischaemic attack (*n* = 8884), or cancer (*n* = 2577) or those with missing values for BMI (*n* = 2), leaving 461,036 participants in the analysis (please note that some participants were excluded for meeting more than one criteria).

### Baseline data collection in CKB

Information on sociodemographic status, smoking [21], alcohol drinking, physical activity [22], medical history and diet [23, 24] were collected by trained health professionals using a laptop-based questionnaire. Each participant provided a 10 ml venous blood sample (with time since last eating or drinking any energy-containing food or beverage recorded). Anthropometry (e.g. body weight, height, waist circumference) [3] and BP [25] were measured following standard protocols. BMI was calculated as weight (kg) divided by height squared (m^2^). In addition, body fat percentage (BF%) was estimated using a TBF-300 monitor (Tanita, Tokyo, Japan). Random blood glucose levels were measured immediately following sample collection using the SureStep Plus System (Johnson & Johnson, New Brunswick, NJ, USA), which provided plasma-equivalent readings and was regularly calibrated with manufacturer’s control solutions. Individuals who did not report a history of diabetes but who had random blood glucose ≥11.1 mmol/l or fasting blood glucose ≥7.0 mmol/l were defined as having screen-detected diabetes. Participants with either screen-detected diabetes or self-reported prior history of physician-diagnosed diabetes were classified as prevalent diabetes and excluded from the present study.

### Dietary assessment

**Fig. 1:**
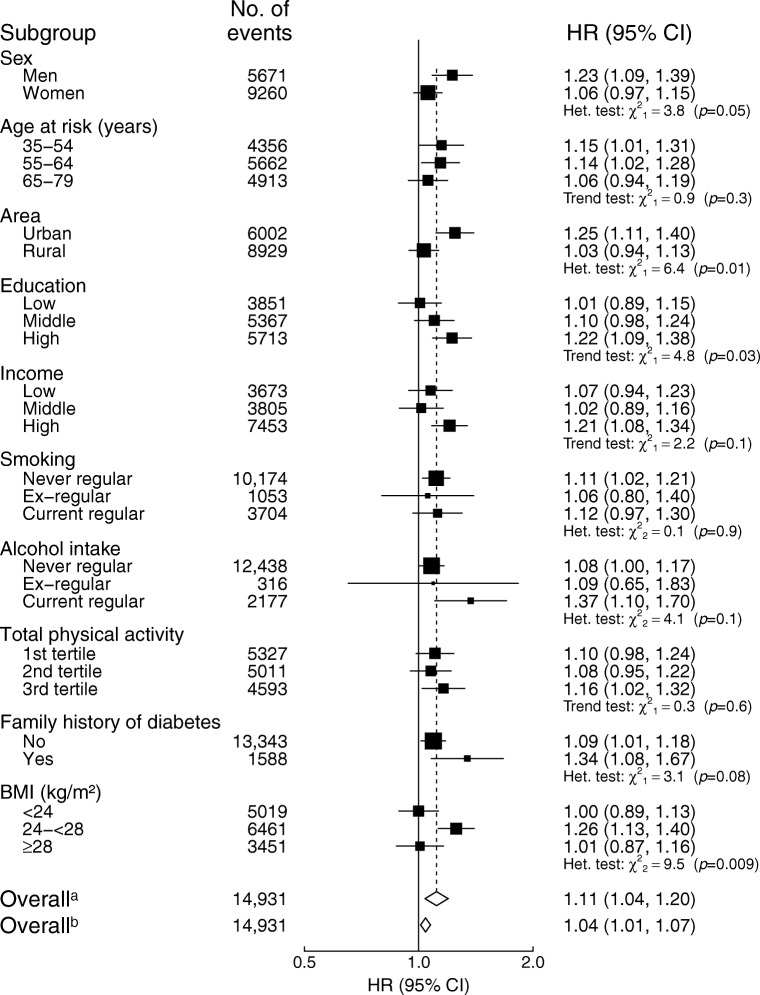
Adjusted HRs (95% CIs) for diabetes per 50 g/day of red meat intake by subgroups. Analyses were stratified by age-at-risk, sex and region, and adjusted for education, income, smoking, alcohol intake, physical activity, consumption of fresh fruit, fish and poultry, family history of diabetes, and BMI. Black squares, HRs (size is inversely proportional to the variance of the log_*e*_ HR); horizontal lines, 95% CIs; white diamonds, overall HRs. ^a^Overall HR per 50 g/day usual red meat intake after correcting for regression dilution bias. ^b^Overall HR per 50 g/day baseline red meat intake before correcting for regression dilution bias. ‘No. of events’ refers to the number of incident diabetes cases in each group. The subscript numbers in the χ^2^ values represent the degrees of freedom

Information on consumption frequency (daily, 4–6 days/week, 1–3 days/week, monthly or never/rarely) of red meat (fresh and processed pork, beef and lamb/mutton), poultry (chicken, duck and goose) and fish (fish and shellfish) was collected using a validated interviewer-administered laptop-based questionnaire asking participants to report their eating habits during the past 12 months. The questionnaire has good reproducibility and relative validity against multiple 24 h recalls (weighted κ was 0.60, 0.61 and 0.75, respectively, for red meat, poultry and fish intake) [26]. In addition, strong positive associations were found between red meat consumption and blood levels of creatinine, total choline and sphingomyelin, and between fish consumption and blood levels of docosahexaenoic acid (DHA), DHA/fatty acid ratio, and total *n*-3 fatty acids (see electronic supplementary material [ESM] Fig. 1).

Following the completion of the baseline survey (2004–2008), 5–6% of the surviving participants were randomly selected to participate in re-surveys in order to understand the long-term variations and measurement errors of various baseline exposures. During the re-survey conducted in 2013–2014 (response rate 76%), the quantity of each food group consumed in addition to the consumption frequency was recorded, allowing us to estimate the usual mean amount consumed (i.e. average intake level during follow-up period) for each baseline exposure category.

### Follow-up for incident diabetes

The vital status of each participant was obtained periodically through China’s Disease Surveillance Points (DSP) system [27] (death registry checked annually against local residential and health insurance records, and by street committees or village administrators). In addition, information on diabetes incidence was collected through linkages with chronic disease registries (for IHD, stroke, cancer and diabetes) and national health insurance claim databases, which provided almost universal (~99%) coverage of all hospitalisations for participants in the study. Both fatal and non-fatal events were coded using ICD-10 (https://icd.who.int/browse10/2014/en) by staff who were blinded to baseline information [20]. For the present study, incident diabetes included all recorded cases (E10-E14) that occurred between the ages of 35 and 79 years. A medical record review of approximately 1000 incidences of diabetes confirmed the validity of diabetes diagnosis (positive predictive value 97%). By 1 January 2017 (global censoring date), only 5276 (~1%) participants were lost to follow-up and they were censored in the prospective analyses.

### Statistical analysis

To ensure an adequate number of diabetes cases in each consumption category for the prospective analyses, individuals were classified into four groups for red meat (daily, 4–6 days/week, 1–3 days/week and <1 day/week) and fish (≥4 days/week, 1–3 days/week monthly and never/rarely) consumption, and three groups for poultry consumption (weekly, monthly and never/rarely) by combining those original categories with less than 5% participants into the adjacent categories.

Means (SDs) or percentages of baseline characteristics were calculated across categories of each dietary exposure, adjusting for age, sex and region, where appropriate, using either multiple linear regression for continuous outcomes or logistic regression for binary outcomes. Cross-sectional associations of each dietary exposure under study with adiposity (BMI, waist circumference and BF%) were examined in men and women separately using multiple linear regression analyses. Adjustments were made for age (continuous variable), region (ten regions), smoking (four categories), alcohol intake (four categories), education (four categories), income (four categories), physical activity (continuous variable) and fresh fruit intake (five categories), and mutual adjustment for intake of the other two exposure variables. Analyses for waist circumference and BF% were additionally adjusted for BMI.

HRs and 95% CIs for diabetes incidence across exposure categories were estimated using Cox proportional hazards models, stratified by age-at-risk (groups of 5 years), sex and region, and adjusted for potential confounders including the above-mentioned covariates and family history of diabetes (dichotomous). Except for fresh fruit [24] and the three dietary exposures under study, no other dietary variables were included in the main models because none were associated with diabetes risk in the current analysis. In model 4, BMI (continuous) was also added in as a covariate. The proportion of diabetes risk explained by BMI was calculated as follows: [(log_*e*_ HR_model3_ − log_*e*_ HR_model4_)/log_*e*_ HR_model3_] × 100%. The mean proportion and associated 95% CIs were obtained through bootstrap techniques with 1000 replications. The ‘floating absolute risk’ method was used to calculate 95% CIs of HRs in all exposure categories (including the reference category), without altering the point estimates. This method allows valid comparisons to be made between any two exposure groups for polychotomous risk factors [28]. We used data from 20,084 participants who attended the re-survey in 2013–2014 to correct for regression dilution bias [29, 30] and quantify the mean usual consumption quantities for each baseline exposure category (ESM Methods). The HR for each 50 g/day of usual red meat, poultry and fish intake was calculated using Cox regression analyses.

Stratified analyses by potential effect modifiers (e.g. sex, region, socioeconomic status [SES] and BMI) were performed and χ^2^ tests for trend and heterogeneity were applied to the log_*e*_ HR and its SE. Comparison of HRs for the first and second halves of the follow-up period revealed no clear evidence of departure from the proportional hazards assumption. Sensitivity analyses were performed by excluding the first 2 years of follow-up or participants with incident cardiovascular disease (CVD) and cancer during follow-up, and by additional adjustment for other dietary factors and other adiposity indices.

All analyses were conducted using SAS (version 9.3, SAS Institute, Cary, NC, USA). Graphs were plotted using R 3.3.2 (https://www.R-project.org/).

## Results

The mean (SD) baseline age of the study participants was 51.2 (10.5) years, 59% were women and 58% resided in rural areas. At baseline, 28.7% of the participants consumed red meat on a daily basis and 17.3% reported <1 day/week consumption (Table 1). Those participants who consumed red meat more frequently were younger, male, urban residents, and had higher education and income levels. They were also more likely to be regular smokers and regular alcohol drinkers. Similar associations between age, sex, SES, and alcohol intake and poultry and fish consumption were also found (ESM Table 1). Except for whole grain and preserved vegetables, consumption of other dietary variables was positively correlated with red meat, poultry and fish consumption, particularly fresh fruit, dairy products and eggs.

**Table 1:** Baseline characteristics of participants by frequency of red meat consumption

Characteristic	Frequency of red meat consumption				Overall (*n* = 461,036)
	<1 day/week (*n* = 79,615)	1–3 days/week (*n* = 164,895)	4–6 days/week (*n* = 84,223)	Daily (*n* = 132,303)	
Usual meat consumption, g/day^a^	23.3	52.0	61.7	71.5	55.1
Mean age (SD), years	54.4 (12.2)	52.2 (10.6)	50.1 (10.7)	48.8 (11.5)	51.2 (10.5)
Women, %	72.1	62.5	55.0	49.2	59.0
Urban, %	12.2	30.8	42.4	74.5	42.3
Education >6 years, %	40.0	47.5	51.4	56.1	49.4
Household income >20,000 yuan/year, %	26.8	37.8	49.1	54.2	42.6
Ever regular smoking, % in men^b^	70.0	73.7	75.0	77.3	74.6
Ever regular alcohol drinking, % in men^b^	21.8	36.5	38.9	44.0	37.2
Frequency of food consumption^c^					
Fish	9.7	7.1	8.7	10.9	8.9
Poultry	25.3	36.3	44.2	33.6	35.1
Fresh fruit	17.0	23.3	31.5	37.2	27.7
Fresh vegetables	91.7	93.7	94.3	97.9	94.6
Preserved vegetables	25.9	19.8	22.1	24.1	22.5
Eggs	14.6	20.8	28.8	29.8	23.8
Dairy products	8.0	9.4	10.8	13.8	10.7
Soybean	7.3	7.9	10.7	12.1	9.5
Whole grain	19.3	14.0	11.3	11.7	13.8
Mean physical activity (SD), MET-h/day	21.4 (14.4)	22.2 (12.4)	22.2 (12.6)	21.5 (13.6)	21.9 (13.9)
Mean BMI (SD), kg/m^2^	23.2 (3.8)	23.4 (3.3)	23.6 (3.3)	23.8 (3.6)	23.5 (3.3)
Mean waist circumference (SD), cm					
Men	79.6 (10.7)	80.9 (9.3)	82.1 (9.4)	82.8 (10.1)	81.6 (9.6)
Women	78.1 (10.7)	78.3 (9.2)	78.6 (9.3)	78.8 (10.1)	78.5 (9.3)
Mean BF% (SD)^d^					
Men	20.4 (6.8)	21.4 (6.0)	22.2 (6.0)	22.7 (6.5)	21.8 (6.2)
Women	31.3 (8.2)	31.7 (7.1)	32.1 (7.1)	32.2 (7.7)	31.8 (7.0)

Values are adjusted for age, sex and region, where appropriate

^a^Crude mean values from second re-survey of randomly selected 20,084 participants without CVD, cancer and diabetes at either baseline or second re-survey

^b^In women, only 3.0% ever regularly smoked and 2.5% ever regularly drunk alcohol

^c^Values indicate the frequency as ‘daily’ for fresh vegetable consumption; ‘≥1 day/week’ for poultry consumption and ‘≥4 days/week (i.e. ‘regular’ for all other food groups)

^d^213 participants had missing values for BF%

MET-h, metabolic equivalent of task hours

The estimated usual mean daily consumption was 55.1 g for red meat, 14.4 g for poultry and 23.1 g for fish, higher in men and in urban areas (ESM Fig. 2). Fish intake showed the largest urban vs rural difference among the three food groups investigated, with Qingdao and Haikou (two coastal urban areas) having the highest mean usual consumption and Gansu and Henan (two inland rural areas) having the lowest (data not shown).

Red meat consumption was positively associated with BMI (ESM Fig. 3), with men and women who consumed red meat daily having a 0.3 and 0.7 kg/m^2^ higher BMI, respectively, compared with those consuming red meat less than once weekly. After accounting for BMI, red meat consumption was not clearly associated with waist circumference and BF%. Likewise, fish consumption was also positively associated with BMI in both men and women, while poultry consumption was positively associated with BMI only in men.

During a mean follow-up of 9 years (~4.5 million person-years), 14,931 incident diabetes cases were recorded at age 35–79 years (incidence rate ~3300 per 10,000). After adjustment for all above-mentioned covariates (including the other two food groups under investigation but not BMI), consumption of red meat and fish was positively associated with risk of diabetes with HR 1.19 (95% CI 1.15, 1.23) and 1.15 (95% CI 1.08, 1.23), respectively, for the highest vs lowest consumption category (Table 2); no such association was noted for consumption of poultry. After correcting for regression dilution bias, each 50 g/day increase in consumption was associated with HR of 1.19 (95% CI 1.11, 1.28) for red meat and an HR of 1.12 (95% CI 1.06, 1.19) for fish. Additional adjustment for BMI attenuated the HR to 1.11 (95% CI 1.04, 1.20) for each 50 g/day increment in usual red meat intake and 1.06 (95% CI 1.00, 1.13) for each 50 g/day fish intake, respectively, corresponding to 36.8% (26.1%, 55.8%) and 46.1% (28.4%, 78.4%) attenuation. Poultry consumption was not associated with diabetes risk before or after adjusting for BMI.

**Table 2:** Risk of new-onset diabetes associated with consumption of red meat, poultry and fish

Consumption	No. of cases	Diabetes risk (95% CI)			
		Model 1^a^	Model 2^b^	Model 3^c^	Model 4^d^
Red meat					
<1 day/week	1612	1.00 (0.94, 1.06)	1.00 (0.94, 1.06)	1.00 (0.94, 1.06)	1.00 (0.94, 1.06)
1–3 days/week	5779	1.08 (1.05, 1.11)	1.09 (1.06, 1.12)	1.08 (1.06, 1.11)	1.06 (1.03, 1.09)
4–6 days/week	3106	1.09 (1.05, 1.13)	1.10 (1.06, 1.14)	1.09 (1.05, 1.13)	1.05 (1.02, 1.09)
Daily	4434	1.21 (1.17, 1.25)	1.20 (1.16, 1.24)	1.19 (1.15, 1.23)	1.12 (1.08, 1.16)
Likelihood ratio χ^2^		39.8	32.8	28.1	11.6
*p*_trend_		<0.0001	<0.0001	<0.0001	0.004
Per 50 g/day at baseline^e^	14,931	1.09 (1.06, 1.12)	1.08 (1.05, 1.11)	1.08 (1.04, 1.11)	1.04 (1.01, 1.07)
Per 50 g/day usual consumption^e^	14,931	1.21 (1.14, 1.30)	1.20 (1.12, 1.29)	1.19 (1.11, 1.28)	1.11 (1.04, 1.20)
Poultry					
Never/rarely	4121	1.00 (0.96, 1.04)	1.00 (0.96, 1.04)	1.00 (0.96, 1.05)	1.00 (0.96, 1.05)
Monthly	6118	1.06 (1.03, 1.08)	1.05 (1.02, 1.07)	1.03 (1.01, 1.05)	1.03 (1.00, 1.05)
Weekly	4692	1.09 (1.05, 1.13)	1.07 (1.03, 1.10)	1.02 (0.99, 1.06)	1.00 (0.97, 1.04)
Likelihood ratio χ^2^		9.7	5.4	1.4	1.8
*p*_trend_		0.002	0.03	0.48	0.93
Per 50 g/day at baseline^e^	14,931	1.08 (1.02, 1.16)	1.06 (0.99, 1.13)	1.01 (0.95, 1.08)	0.99 (0.92, 1.05)
Per 50 g/day usual consumption^e^	14,931	1.23 (1.06, 1.42)	1.16 (1.00, 1.35)	1.04 (0.89, 1.21)	0.96 (0.83, 1.12)
Fish					
Never/rarely	3515	1.00 (0.94, 1.06)	1.00 (0.94, 1.06)	1.00 (0.94, 1.07)	1.00 (0.94, 1.07)
Monthly	3811	1.03 (0.99, 1.06)	1.02 (0.99, 1.06)	1.00 (0.96, 1.03)	0.97 (0.94, 1.01)
1–3 days/week	6225	1.08 (1.05, 1.11)	1.07 (1.04, 1.10)	1.04 (1.01, 1.07)	1.00 (0.97, 1.03)
Regular	1380	1.22 (1.15, 1.30)	1.19 (1.12, 1.27)	1.15 (1.08, 1.23)	1.06 (1.00, 1.13)
Likelihood ratio χ^2^		25.0	18.8	15.2	5.6
*p*_trend_		<0.0001	<0.0001	0.002	0.14
Per 50 g/day at baseline^e^	14,931	1.10 (1.06, 1.14)	1.09 (1.05, 1.13)	1.08 (1.04, 1.12)	1.04 (1.00, 1.08)
Per 50 g/day usual consumption^e^	14,931	1.15 (1.09, 1.22)	1.14 (1.07, 1.20)	1.12 (1.06, 1.19)	1.06 (1.00, 1.13)

The likelihood ratio χ^2^values indicate the strength of the associations of main exposure variable with diabetes risk. A larger χ^2^ indicates a stronger association and a decrease in the χ^2^ indicates that the association is attenuated after additional adjustment for newly added variables

^a^Model 1: stratified by age-at-risk, sex and region

^b^Model 2: as for model 1, additionally adjusted for education, income, smoking, alcohol consumption, physical activity, family history of diabetes, and fresh fruit consumption

^c^Model 3: as for model 2, additionally adjusted for the other two main dietary exposure variables listed in the table

^d^Model 4: as for model 3, additionally adjusted for BMI

^e^The mean amount consumed at the second re-survey was used to estimate the usual consumption level for each group; baseline consumption level was estimated using daily consumption portion at the second re-survey multiplied by the consumption frequency at baseline

**Fig. 2:**
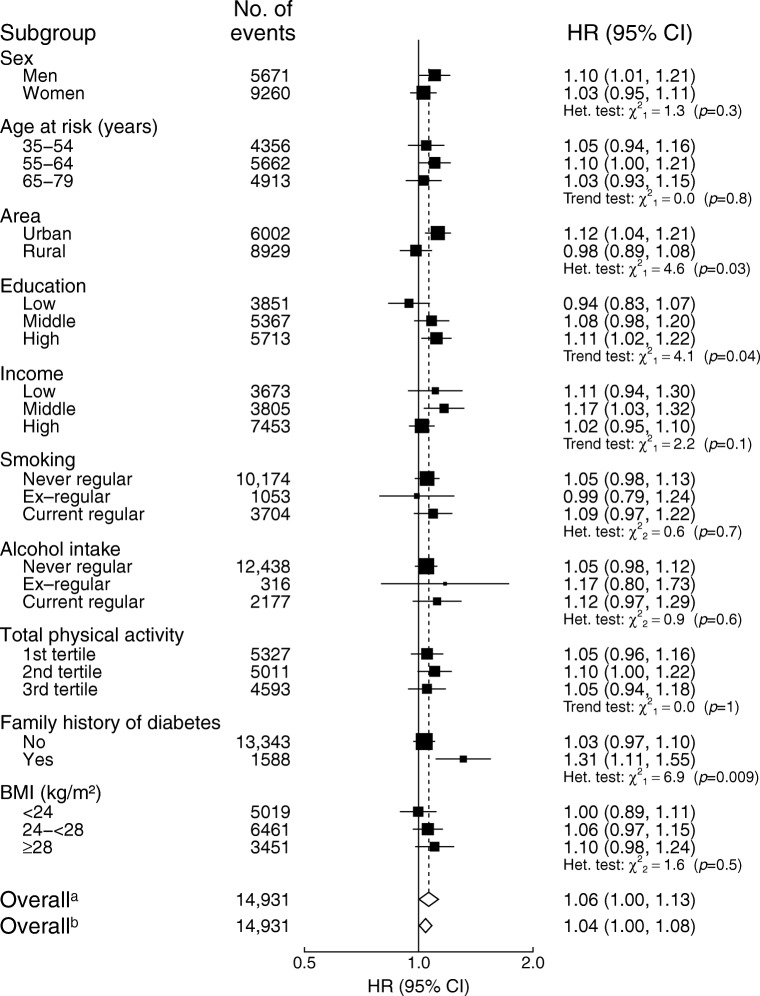
Adjusted HRs (95% CIs) for diabetes per 50 g/day of fish intake by subgroups. Analyses were stratified by age-at-risk, sex and region, and adjusted for education, income, smoking, alcohol intake, physical activity, consumption of fresh fruit, red meat and poultry, family history of diabetes, and BMI. Black squares, HRs (size is inversely proportional to the variance of the log_*e*_ HR); horizontal lines, 95% CIs; white diamonds, overall HRs. ^a^Overall HR per 50 g/day usual fish intake after correcting for regression dilution bias. ^b^Overall HR per 50 g/day baseline fish intake before correcting for regression dilution bias. ‘No. of events’ refers to the number of incident diabetes cases in each group. The subscript numbers in the χ^2^ values represent the degrees of freedom

The association of red meat intake with diabetes was more pronounced in men than in women (HR 1.23 [95% CI 1.09, 1.39] vs HR 1.06 [95% CI 0.97, 1.15] per 50 g/day usual consumption) and was more pronounced in urban areas than in rural areas (HR 1.25 [95% CI 1.11, 1.40] vs HR 1.03 [95% CI 0.94, 1.13]) (*p*_heterogeneity_ = 0.05 and 0.01, respectively; Fig. 1). In addition, the association was stronger in those with higher education level (*p*_trend_ = 0.03), those who were current alcohol drinkers and those with family history of diabetes, although the heterogeneity test was not statistically significant for the latter two. BMI significantly modified the association between red meat consumption and diabetes risk; the association was only significant in the overweight group (not the other two groups). Similarly, the association between fish consumption and risk of diabetes was stronger in residents of urban vs rural areas, in those with higher education and in those with a family history of diabetes (Fig. 2).

**Fig. 3:**
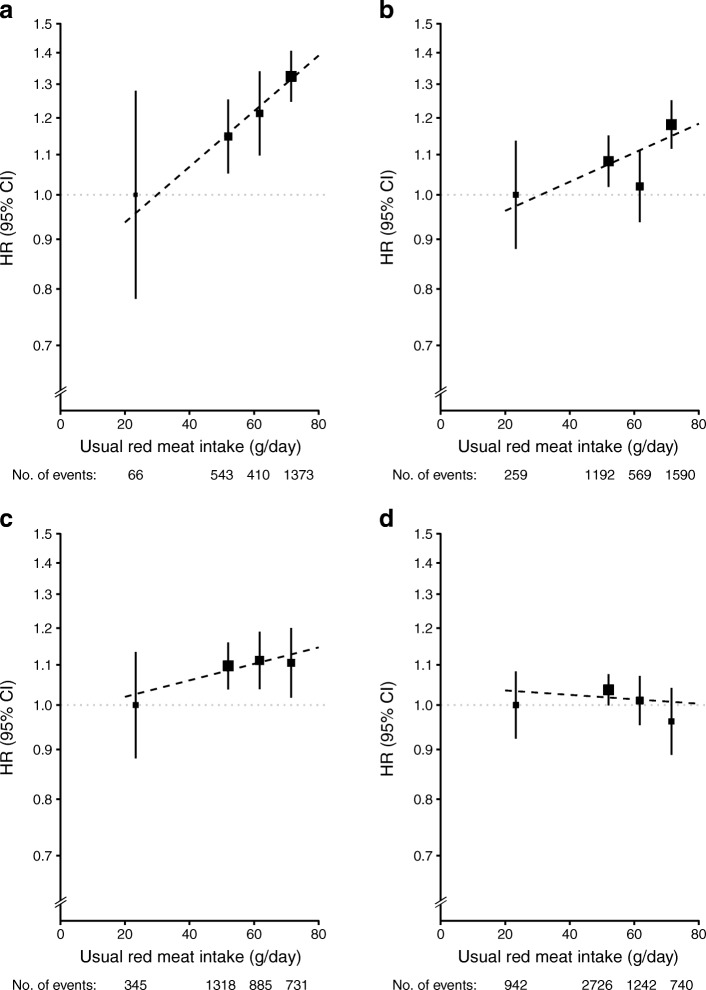
Adjusted HRs (95% CIs) for diabetes associated with red meat intake, by sex and area. (**a**) Urban men. (**b**) Urban women. (**c**) Rural men. (**d**) Rural women. Analyses were stratified by age-at-risk and region and adjusted for education, income, smoking, alcohol intake, physical activity, consumption of fresh fruit, fish and poultry, family history of diabetes, and BMI. The *y* axis was plotted on a log_*e*_ scale with the lowest consumption group as reference category. Black squares, HRs (size is inversely proportional to the variance of the log_*e*_ HR); vertical lines, 95% CIs; dashed diagonal lines, linear associations between red meat consumption diabetes risk

**Fig. 4:**
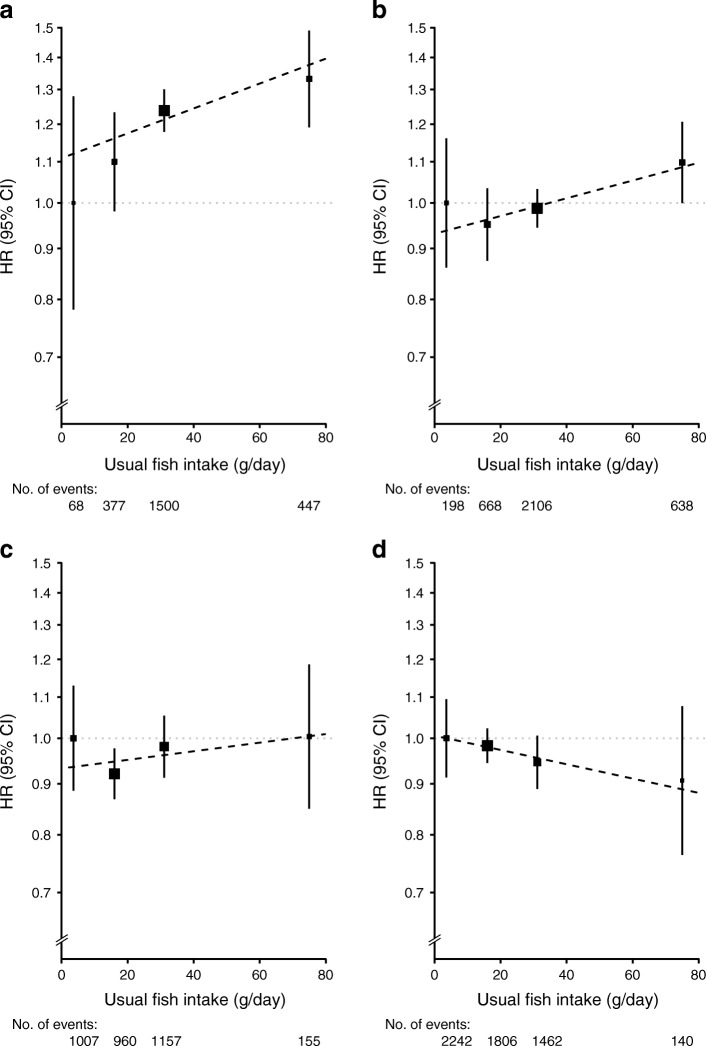
Adjusted HRs (95% CIs) for risk of diabetes associated with fish intake, by sex and area. (**a**) Urban men. (**b**) Urban women. (**c**) Rural men. (**d**) Rural women. Analyses were stratified by age-at-risk and region, where appropriate, and adjusted for education, income, smoking, alcohol intake, physical activity, consumption of fresh fruit, red meat and poultry, family history of diabetes, and BMI. The *y* axis was plotted on a log_*e*_ scale with the lowest consumption group as reference category. Black squares, HRs (size is inversely proportional to the variance of the log_*e*_ HR); vertical lines, 95% CIs; dashed diagonal lines, linear associations between fish consumption diabetes risk

Stratified analyses by sex and area (urban vs rural) showed that both red meat and fish were positively associated with diabetes risk in urban men and women, but not in those from rural areas (Figs 3, 4). Compared with participants who reported consumption of red meat on <1 day/week (the lowest group) at baseline, daily consumption (the highest group) was associated with an HR (95% CI) of 1.32 (1.25, 1.41) in men from urban areas, 1.18 (1.12, 1.25) in women from urban areas, 1.11 (1.02, 1.20) in men from rural areas and 0.96 (0.89, 1.04) in women from rural areas. For each 50 g/day increment in usual red meat intake, the corresponding HR (95% CI) was 1.42 (1.15, 1.74), 1.18 (1.03, 1.36), 1.11 (0.95, 1.30) and 0.98 (0.88, 1.10), respectively, in these four subgroups (Fig. 3). For fish consumption, the HR (95% CI) for diabetes risk in men and women from urban areas and men and women from rural areas reporting the highest consumption was 1.33 (1.19, 1.49), 1.10 (1.00, 1.21), 1.00 (0.85, 1.19) and 0.91 (0.76, 1.08), respectively, compared with those who never or rarely ate fish (Fig. 4). Each 50 g/day increase in usual consumption of fish was associated with an HR (95% CI) of 1.15 (1.02, 1.30), 1.11 (1.01, 1.23), 1.04 (0.91, 1.20) and 0.92 (0.81, 1.05), respectively, (Fig. 4).

Across five urban and five rural areas, the association between red meat intake and diabetes risk varied a little but there was significant heterogeneity across five individual urban areas for fish intake in relation to diabetes risk (ESM Fig. 4).

Sensitivity analyses, including the exclusion of the first 2 years of follow-up, additional adjustment for other dietary factors (i.e. egg, dairy products, soybean, fresh and preserved vegetables, and whole grain staple foods) and other adiposity indices (i.e. waist circumference and BF%), and exclusion of participants who developed CVD and cancer during follow-up, did not materially alter the main results described above (ESM Table 2).

## Discussion

In this large prospective study of Chinese adults, consumption of both red meat and fish (but not poultry) was positively associated with the risk of developing diabetes, with each 50 g/day increase in usual intake associated with 11% and 6% higher risk, respectively. These associations were independent of other dietary factors and adiposity and were more pronounced in urban areas than rural areas and in men than in women.

Red meat is a major source of valuable proteins, essential amino acids, vitamins (e.g. vitamin B_12_) and minerals (e.g. zinc and iron) [31]. Consumption of red meat, however, has been associated with higher risks of several chronic diseases, including diabetes, in western populations. In two large meta-analyses of prospective cohort studies involving >442,000 adults and >17,000 individuals with diabetes, each 100 g/day increase in red meat consumption was associated with 13% and 19% higher risk of diabetes, respectively [6, 7]. To date, only one prospective study from China, involving ~75,000 middle-aged women and ~2000 individuals with diabetes, has been published on this topic, reporting no clear association between red meat consumption and risk of diabetes (HR 0.94 [95% CI 0.80, 1.10] for top vs bottom quintile) [11]. By contrast, in a similar-sized prospective study of Chinese adults living in Singapore (57% women), the HR (95% CI) for diabetes comparing highest vs lowest consumption quartile was 1.23 (1.14, 1.33) [32]. The present study included more incidences of diabetes than these two previous Chinese studies combined and found a statistically significant positive association between red meat consumption and diabetes, particularly in men and women living in urban areas. Moreover, our study findings showed that over one-third of the positive association could be explained by adiposity, which is the most important risk factor for diabetes [3].

The observed clear patterns of effect modifications by sex, area (urban vs rural), and education level suggest a plausible mediating role of iron overload in the association between red meat intake and diabetes [9]. That is because women, participants living in rural areas and people with lower SES tend to have relatively lower levels of body iron stores [33] and, as such, eating red meat may not lead to iron overload and diabetes. In addition, the much stronger associations seen in current alcohol drinkers and participants with a family history of diabetes might also relate to a higher level of iron storage because drinking alcohol and carrying certain genetic variants could lead to a higher rate of dietary iron absorption [34, 35]. However, directly measured iron status was not currently available in our study and future studies with this information are required to confirm or refute this hypothesis.

In the present study population, the mean level of consumption was much lower for poultry than red meat and its null association with diabetes is consistent with the overall available evidence to date [6]. Although the consumption of fish is generally recommended by most dietary guidelines for prevention of CVD [36], existing evidence overall does not support any clear beneficial association with diabetes. For example, in a meta-analysis of 13 cohort studies involving 481,489 participants and 20,830 cases of incident diabetes, fish intake was not in an overall significant association with diabetes incidence (RR 1.12 [95% CI 0.94, 1.34] per 100 g/day), with substantial heterogeneity across different cohorts [17]. However, in separate analyses by geographic location of study populations, fish intake was inversely associated with risk of diabetes (HR 0.89 [95% CI 0.98, 0.81] per 100 g/day intake) in five Asian cohorts (including two from China), involving ~7100 diabetes cases, but positively associated with diabetes risk (HR 1.38 [95% CI 1.13, 1.70]) in eight North American/European cohorts (~13,700 cases). Though less extreme, our findings, particularly among participants residing in urban areas, were broadly consistent with those from previous western studies conducted in North America and Europe [37, 38]. The potential mechanisms underlying this positive association are unclear but both iron [39, 40] and environmental contaminants (e.g. mercury) in fish might play a role [41]. In addition, higher consumption of animal-sourced foods (i.e. red meat and fish) is very likely associated with affluence-related dietary patterns, which may increase the risk of diabetes in China [42].

The present study has several major strengths. First, the large sample size in the CKB allows us to exclude diagnosed and undiagnosed diabetes as well as major chronic diseases (i.e. CVD and cancer) at baseline, thus limiting the potential influence of reverse causality. Second, the analyses controlled for a wide range of potential confounding factors and regression dilution bias caused by long-term variation and inevitable measurement errors in self-reported dietary exposure variables [43]. More importantly, the main exposure variables in the present study had good reproducibility and validity.

The study also has several limitations. First, we collected outcome information through linkage with hospitalisation records, so some non-hospitalised incidences of diabetes may have been missed. However, we have previously observed that diabetes prevalence based on the CKB re-survey population was reasonably consistent with nationally representative surveys [3]. On the other hand, such under-reporting in outcome measures would most likely be non-differential and thus not overestimate risk estimates. Second, our dietary questionnaire is relatively simple, collecting consumption data for only some of the major food groups instead of individual food items. Hence, it was not possible to adjust for total energy intake and other specific dietary factors (e.g. saturated fat, salt and dietary fibre). However, total energy intake should not play a major role in the observed associations because our main analyses were adjusted for both BMI and physical activity, which together could be considered as a good proxy for total energy intake. Third, we were unable to distinguish between unprocessed and processed red meat or between different types of red meat (i.e. pork, beef or lamb) and fish (e.g. fatty fish or lean fish). However, the nationally representative nutrition survey showed that unprocessed pork accounts for ~80% of total red meat consumption in China [44]. Fourth, the observed heterogeneities across BMI categories (for red meat) and across regions could not be properly explained. Last, as in all observational studies, the possibility of residual confounding cannot be ruled out and causality cannot be automatically assumed.

In summary, in this large study of a Chinese adult population, higher consumption of red meat was associated with higher risk of new-onset diabetes and this association was only partly explained by adiposity. The association appeared to be stronger in men, participants residing in urban areas and those with a higher education level. Our data do not support the inverse association between fish consumption and diabetes risk previously reported in some Asian studies. Further studies are warranted to understand the exact mechanisms linking red meat and fish consumption with increased risk of diabetes.

## Electronic supplementary material

ESM (PDF 1220 kb)

## Data Availability

Details of how to access the CKB data and rules of CKB data release are available from www.ckbiobank.org/site/Data+Access.

## Abbreviations

BF%: Body fat percentage
CKB: China Kadoorie Biobank
CVD: Cardiovascular disease
DHA: Docosahexaenoic acid
IHD: Ischaemic heart disease
SES: Socioeconomic status
