# Supplementary material for: Red meat, poultry and fish consumption and risk of diabetes: a 9 year prospective cohort study of the China Kadoorie Biobank
Source: Diabetologia. 2020 Jan 22;63(4):767–79. doi: 10.1007/s00125-020-05091-x (PMC7054352; doi:10.1007/s00125-020-05091-x)
Supplement: Supplementary file 1 — (PDF 1220 kb) [file 125_2020_5091_MOESM1_ESM.pdf]

ESM Methods: Correction of regression dilution bias

We used resurvey data collected during 2013-2014 resurvey to estimate usual consumption amount and to account for regression dilution bias.

- We first calculate the mean consumption amount for participants in each baseline consumption group. Taking red meat consumption as an example, among the 20,084 participants who attended the resurvey, 5836 of them reported a daily consumption at baseline. At resurvey, 66.1% reported a daily consumption and, due to either long-term variation or reporting errors, 10.6% reported 4-6 days/week, 15.3% reported 1-3 days/week, 3.1% reported monthly and 5.0% reported never/rarely consumption. Their overall mean consumption amount at the resurvey was 71.5 g/day. We therefore assigned this value to everybody in the daily consumption group (n=132,303) in the main analysis.
- Similarly, 61.7, 52.0 and 23.3 g/day respectively was assigned to 4-6 days/week, 1-3 days/week and <1 day/week baseline red meat consumption group.
- The usual fish consumption amount value assigned to each baseline group was 75.0, 31.1, 16.0 and 3.6 g/day respectively and the usual poultry consumption amount value was 24.8, 13.2 and 7.7 g/day respectively.
- After assigning these consumption amount to each individual participants involved in the main analysis, we put this consumption amount in the Cox regression model to calculate the HRs (and 95% CI) of each 50 g/day usual consumption.

ESM Table 1. Baseline characteristics of participants by frequency of poultry and fish consumption

	Frequency of poultry consumption*			Frequency of fish consumption*			
	Never/rarely (n=170,231)	Monthly (n=161,638)	Weekly [a] (n=129,167)	Never/rarely (n=150,511)	Monthly (n=96,812)	1-3 days/week (n=172,560)	Regular [a] (n=41,153)
Usual poultry/fish consumption, g/day [b]	7.7	13.2	24.8	3.6	16.0	31.1	75.0
Mean age (SD), years	53.4 (12.6)	51.1 (11.0)	48.5 (12.0)	53.9 (17.1)	51.2 (11.2)	49.5 (13.3)	48.4 (12.6)
Women, %	67.4	58.8	48.2	69.4	58.0	52.8	48.9
Urban, %	18.3	42.7	73.3	5.7	36.3	67.5	84.3
Education > 6 years, %	39.7	49.9	61.5	35.1	48.4	58.6	64.9
Household income >20,000 yuan/year, %	30.1	43.9	57.7	24.3	41.7	54.3	62.9
Ever regular smoking, % in men [c]	75.4	74.2	73.9	74.4	74.5	73.9	78.0
Ever regular alcohol drinking, % in men [c]	31.5	37.3	43.3	28.2	36.4	41.7	49.7
Frequency of food consumption [d]							
Red meat	16.5	28.1	45.6	14.8	28.6	37.5	42.7
Fish	6.2	8.8	12.6	-	-	-	-
Poultry	-	-	-	19.4	59.6	34.8	35.4
Fresh fruit	17.8	27.1	41.4	13.7	23.5	37.0	49.4
Fresh vegetables	92.6	96.1	95.5	93.2	95.1	95.0	97.4
Preserved vegetables	21.9	23.0	22.7	20.9	22.5	23.4	24.3
Eggs	17.0	25.3	30.8	15.6	22.1	27.8	40.6
Dairy products	6.8	10.4	16.2	4.7	9.0	14.2	21.7
Soybean	6.2	9.7	13.6	6.2	8.5	10.6	19.3
Whole grain	14.0	11.6	16.1	15.3	10.5	12.6	20.8
Mean physical activity (SD), MET-hr/day	22.5 (14.8)	21.9 (12.9)	21.0 (14.1)	23.1 (20.0)	21.8 (13.1)	21.2 (15.6)	20.5 (14.8)
Mean BMI (SD), kg/m^2	23.4 (3.9)	23.5 (3.4)	23.8 (3.8)	23.2 (5.3)	23.5 (3.5)	23.7 (4.1)	24.0 (3.9)
Mean waist circumference (SD), cm							
Men	80.3 (11.2)	81.6 (9.6)	82.9 (10.5)	79.4 (14.8)	81.4 (9.7)	82.8 (11.5)	83.9 (10.9)
Women	78.4 (10.9)	78.5 (9.6)	78.5 (10.6)	78.0 (14.9)	78.6 (9.8)	78.7 (11.7)	79.2 (11.1)
Mean body fat percentage (SD), % [e]							
Men	21.0 (7.2)	21.9 (6.1)	22.7 (6.7)	20.5 (9.5)	21.7 (6.2)	22.6 (7.3)	23.3 (7.0)
Women	31.7 (8.4)	31.9 (7.4)	32.0 (8.1)	31.4 (11.4)	31.8 (7.5)	32.1 (9.0)	32.6 (8.5)

* Values were adjusted for age, sex and region, where appropriate

a The weekly poultry consumption group contained 122,994 (26.7% of total participants) who consumed poultry 1-3 days/week, 4189 (0.9%) 4-6 days/week and 1993 (0.4%) daily. The regular fish consumption group contained 28,506 (6.2%) who consumed fish 4-6 days/week and 12,649 (2.7%) daily.

b Crude mean values from 2nd resurvey of randomly selected 20,084 participants without CVD, cancer and diabetes at either baseline or 2nd resurvey.

c In women, only 3.0% ever regularly smoked and 2.5% ever regularly drunk alcohol.

d Values indicate the frequency as "daily" for fresh vegetable consumption; "≥1 day/week" for poultry consumption and "≥ 4 days/week, i.e. regular" for all other food groups.

e 213 participants had missing values for body fat percentage.

MET-h: Metabolic equivalent of tasks hours

ESM Table 2. Adjusted HRs for risk of new-onset diabetes associated with consumption of red meat, poultry and fish with different exclusions and adjustments

	Exclude first 2 years follow-up	Additionally adjust for eggs and dairy products	Additionally adjust for other dietary variables*	Additionally adjust for WC	Additionally adjust for BF%	Exclude incident CVD and incident cancer during follow-up
**Red Meat**						
< 1 day/week	1.00 (0.94, 1.06)	1.00 (0.94, 1.06)	1.00 (0.94, 1.06)	1.00 (0.94, 1.06)	1.00 (0.94, 1.06)	1.00 (0.94, 1.06)
1-3 days/week	1.07 (1.04, 1.10)	1.07 (1.04, 1.10)	1.06 (1.03, 1.09)	1.06 (1.03, 1.09)	1.07 (1.04, 1.10)	1.06 (1.03, 1.09)
4-6 days/week	1.05 (1.01, 1.09)	1.06 (1.02, 1.10)	1.05 (1.02, 1.09)	1.05 (1.02, 1.09)	1.05 (1.01, 1.08)	1.04 (1.00, 1.08)
Daily	1.14 (1.09, 1.18)	1.12 (1.08, 1.16)	1.11 (1.07, 1.15)	1.12 (1.08, 1.16)	1.11 (1.07, 1.15)	1.10 (1.06, 1.14)
**Poultry**						
Never/rarely	1.00 (0.95, 1.05)	1.00 (0.96, 1.05)	1.00 (0.96, 1.05)	1.00 (0.96, 1.05)	1.00 (0.96, 1.05)	1.00 (0.95, 1.05)
Monthly	1.02 (0.99, 1.04)	1.03 (1.01, 1.05)	1.03 (1.01, 1.05)	1.03 (1.01, 1.05)	1.03 (1.00, 1.05)	1.02 (0.99, 1.04)
Weekly	1.00 (0.96, 1.04)	1.01 (0.97, 1.05)	1.01 (0.97, 1.05)	1.01 (0.97, 1.05)	1.00 (0.96, 1.04)	1.00 (0.96, 1.04)
**Fish**						
Never/rarely	1.00 (0.94, 1.07)	1.00 (0.94, 1.07)	1.00 (0.94, 1.07)	1.00 (0.94, 1.07)	1.00 (0.94, 1.07)	1.00 (0.94, 1.07)
Monthly	0.96 (0.93, 1.00)	0.98 (0.94, 1.01)	0.98 (0.94, 1.01)	0.97 (0.94, 1.01)	0.97 (0.94, 1.00)	0.96 (0.93, 1.00)
1-3 days/week	1.00 (0.97, 1.03)	1.00 (0.98, 1.03)	1.01 (0.98, 1.04)	1.00 (0.97, 1.03)	0.99 (0.97, 1.02)	0.99 (0.96, 1.02)
Regularly	1.09 (1.02, 1.16)	1.06 (1.00, 1.13)	1.07 (1.00, 1.14)	1.06 (1.00, 1.13)	1.05 (0.99, 1.12)	1.06 (0.99, 1.13)

Analyses were stratified by age-at-risk, sex and region and were adjusted, minimally, for education, income, smoking, alcohol consumption, physical activity, family history of diabetes, fresh fruit consumption, BMI, and the other two dietary variables listed in the Table.

*Including soybean, fresh and preserved vegetables, and whole grain staple foods.

ESM Table 2. Adjusted HRs for risk of new-onset diabetes associated with consumption of red meat, poultry and fish with different exclusions and adjustments

	Exclude first 2 years follow-up	Additionally adjust for eggs and dairy products	Additionally adjust for other dietary variables*	Additionally adjust for WC	Additionally adjust for BF%	Exclude incident CVD and incident cancer during follow-up
**Red Meat**						
< 1 day/week	1.00 (0.94, 1.06)	1.00 (0.94, 1.06)	1.00 (0.94, 1.06)	1.00 (0.94, 1.06)	1.00 (0.94, 1.06)	1.00 (0.94, 1.06)
1-3 days/week	1.07 (1.04, 1.10)	1.07 (1.04, 1.10)	1.06 (1.03, 1.09)	1.06 (1.03, 1.09)	1.07 (1.04, 1.10)	1.06 (1.03, 1.09)
4-6 days/week	1.05 (1.01, 1.09)	1.06 (1.02, 1.10)	1.05 (1.02, 1.09)	1.05 (1.02, 1.09)	1.05 (1.01, 1.08)	1.04 (1.00, 1.08)
Daily	1.14 (1.09, 1.18)	1.12 (1.08, 1.16)	1.11 (1.07, 1.15)	1.12 (1.08, 1.16)	1.11 (1.07, 1.15)	1.10 (1.06, 1.14)
**Poultry**						
Never/rarely	1.00 (0.95, 1.05)	1.00 (0.96, 1.05)	1.00 (0.96, 1.05)	1.00 (0.96, 1.05)	1.00 (0.96, 1.05)	1.00 (0.95, 1.05)
Monthly	1.02 (0.99, 1.04)	1.03 (1.01, 1.05)	1.03 (1.01, 1.05)	1.03 (1.01, 1.05)	1.03 (1.00, 1.05)	1.02 (0.99, 1.04)
Weekly	1.00 (0.96, 1.04)	1.01 (0.97, 1.05)	1.01 (0.97, 1.05)	1.01 (0.97, 1.05)	1.00 (0.96, 1.04)	1.00 (0.96, 1.04)
**Fish**						
Never/rarely	1.00 (0.94, 1.07)	1.00 (0.94, 1.07)	1.00 (0.94, 1.07)	1.00 (0.94, 1.07)	1.00 (0.94, 1.07)	1.00 (0.94, 1.07)
Monthly	0.96 (0.93, 1.00)	0.98 (0.94, 1.01)	0.98 (0.94, 1.01)	0.97 (0.94, 1.01)	0.97 (0.94, 1.00)	0.96 (0.93, 1.00)
1-3 days/week	1.00 (0.97, 1.03)	1.00 (0.98, 1.03)	1.01 (0.98, 1.04)	1.00 (0.97, 1.03)	0.99 (0.97, 1.02)	0.99 (0.96, 1.02)
Regularly	1.09 (1.02, 1.16)	1.06 (1.00, 1.13)	1.07 (1.00, 1.14)	1.06 (1.00, 1.13)	1.05 (0.99, 1.12)	1.06 (0.99, 1.13)

Analyses were stratified by age-at-risk, sex and region and were adjusted, minimally, for education, income, smoking, alcohol consumption, physical activity, family history of diabetes, fresh fruit consumption, BMI, and the other two dietary variables listed in the Table.

*Including soybean, fresh and preserved vegetables, and whole grain staple foods.

ESM Fig. 1. Associations of self-reported consumption of red meat (a-c) and fish (d-f) with blood biomarkers

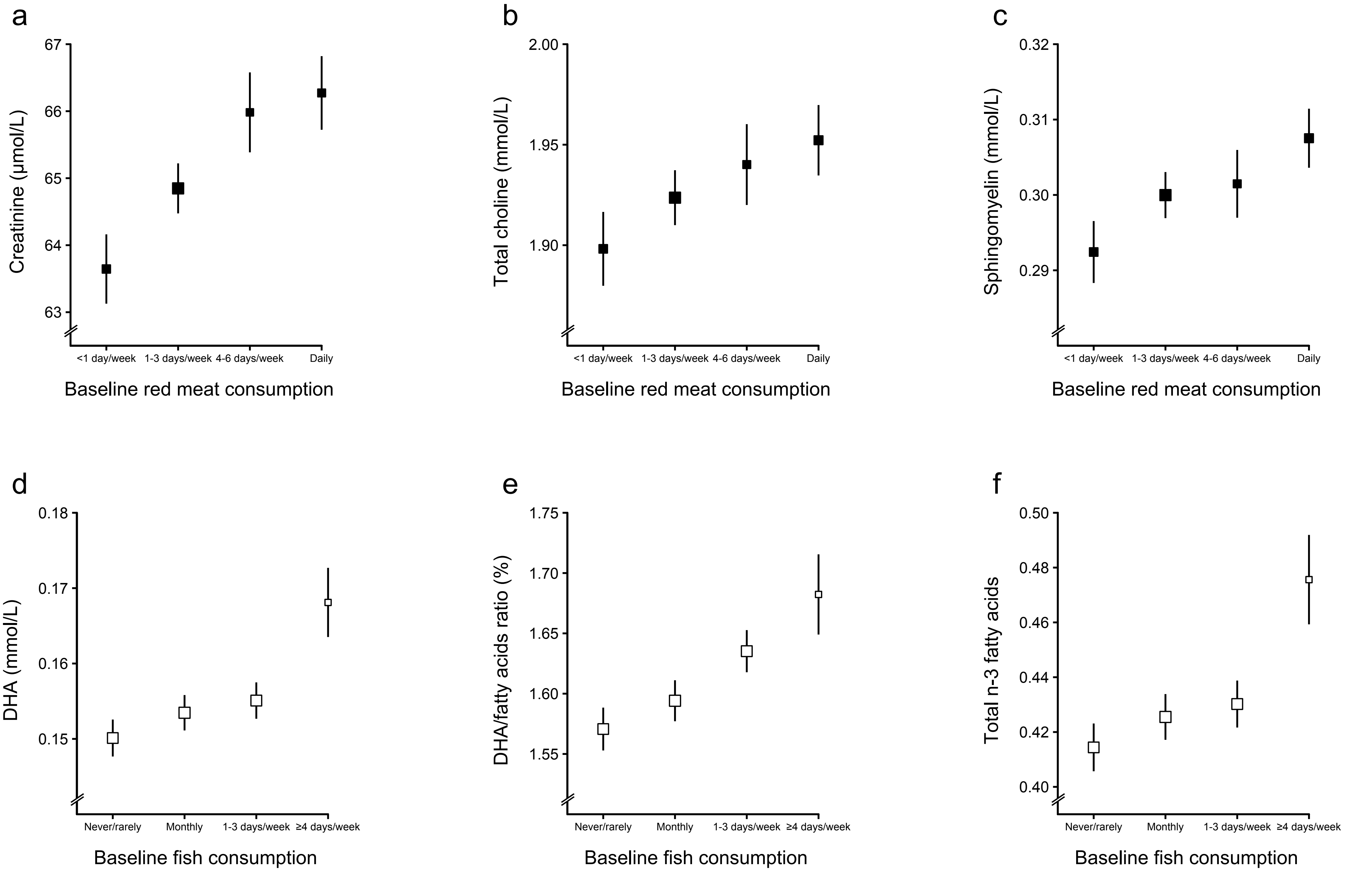

Analyses were adjusted for age, sex, region, education, income, smoking, alcohol, BMI, physical activity, fasting hours, CVD status, and the consumption of fresh fruit, poultry, eggs, dairy products, and, where appropriate, red meat and fish. For analyses on creatinine, Cystatin C was also adjusted for in order to take into account the potential influence of renal function. P for trend<0.0001 for all.
n = 16,160 for (a); n = 4,386 for (b) and (c); n = 4,374 for (d), (e) and (f).

ESM Fig. 2 Mean consumption (g/day) of red meat, poultry and fish by sex and area at resurvey in 2013-2014

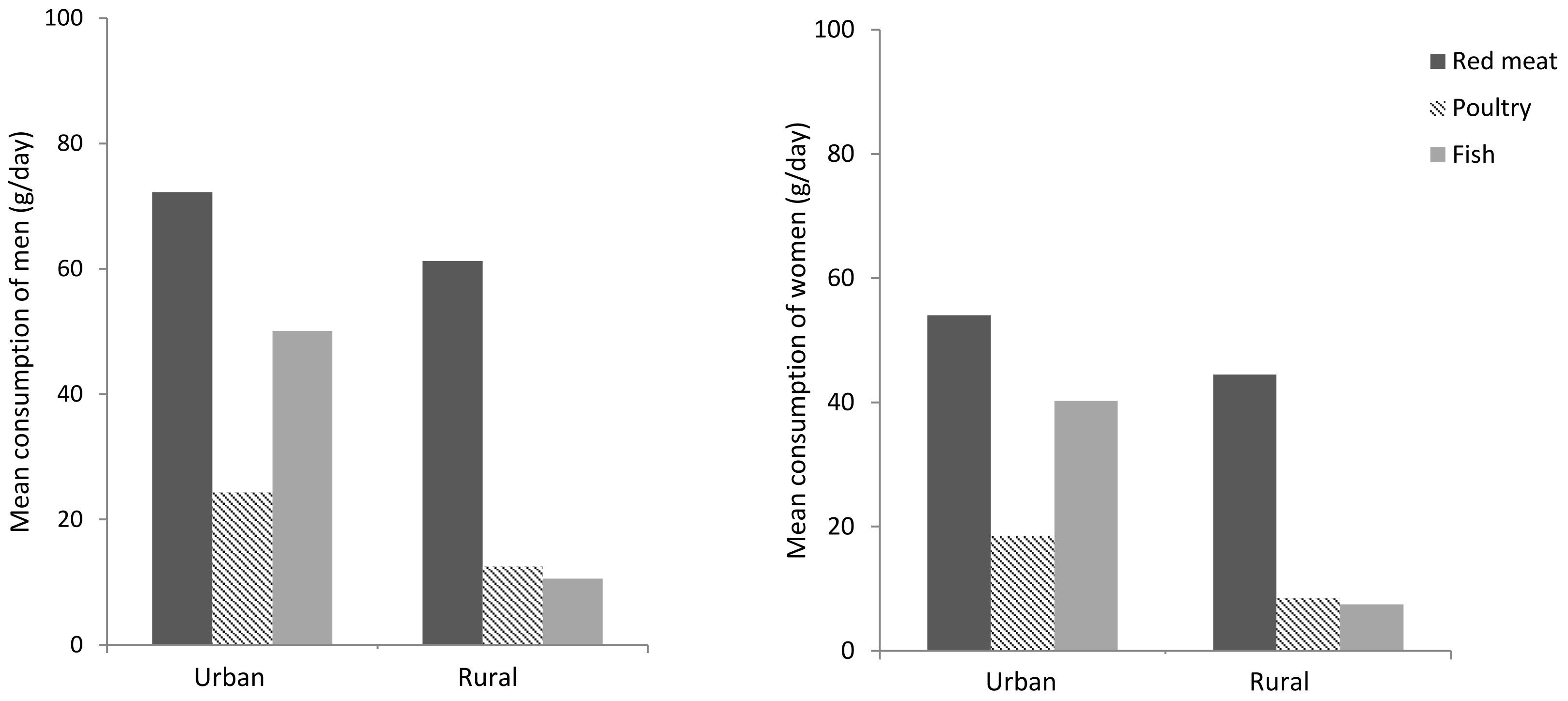

ESM Fig. 3. Adjusted mean levels of adiposity indices by baseline consumption of red meat, fish and poultry in men (M) and women (F)

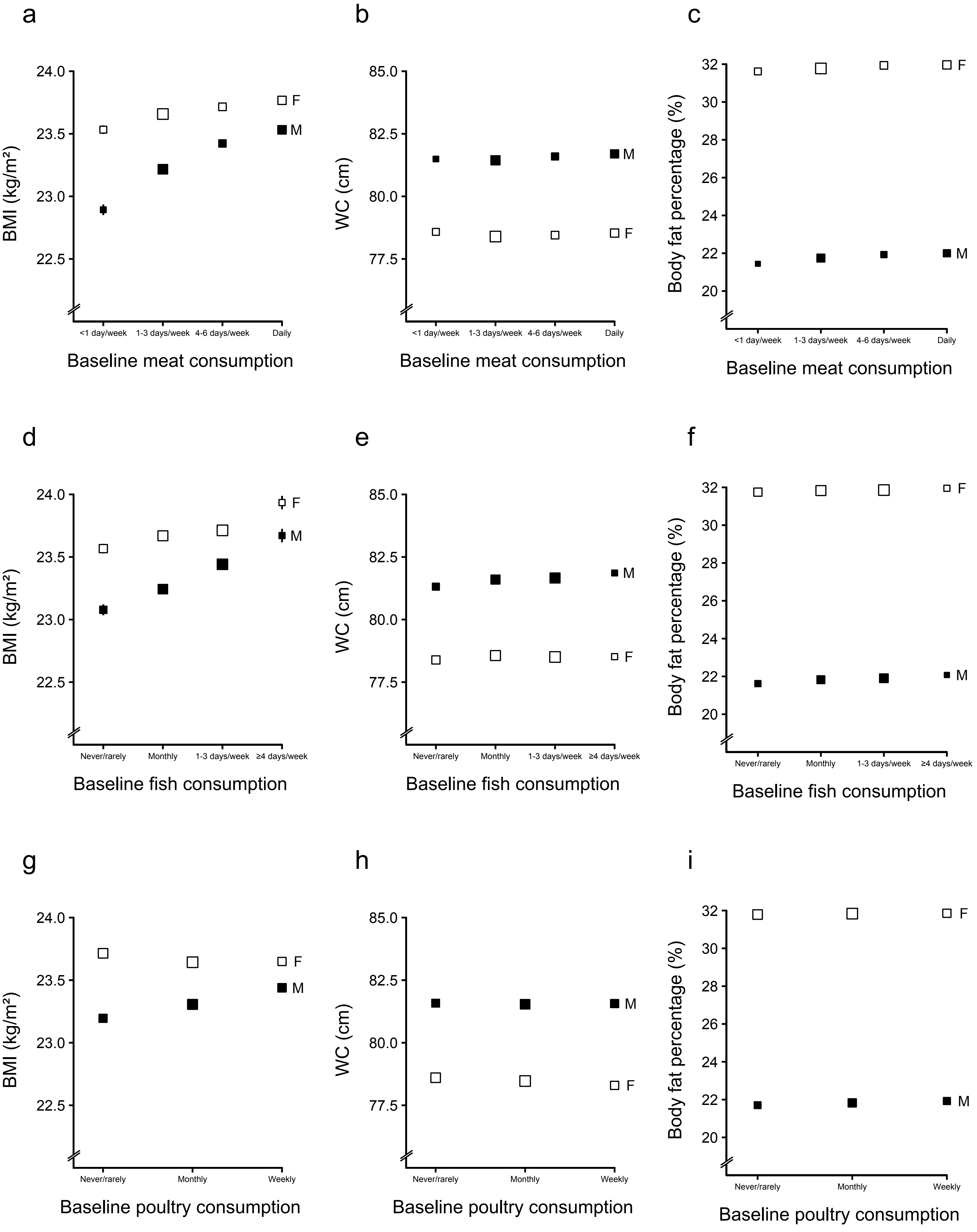

Analyses were adjusted for age, region, smoking, alcohol, education, income, physical activity, and the consumption of fresh fruit, poultry, and, where appropriate, red meat and fish. Analyses in b, c, e, f, h, and i were additionally adjusted for BMI.
Solid squares represent men and open squares represent women.

ESM Fig. 4. Adjusted HRs (95% CIs) for diabetes per 50 g/day usual red meat and fish consumption by region

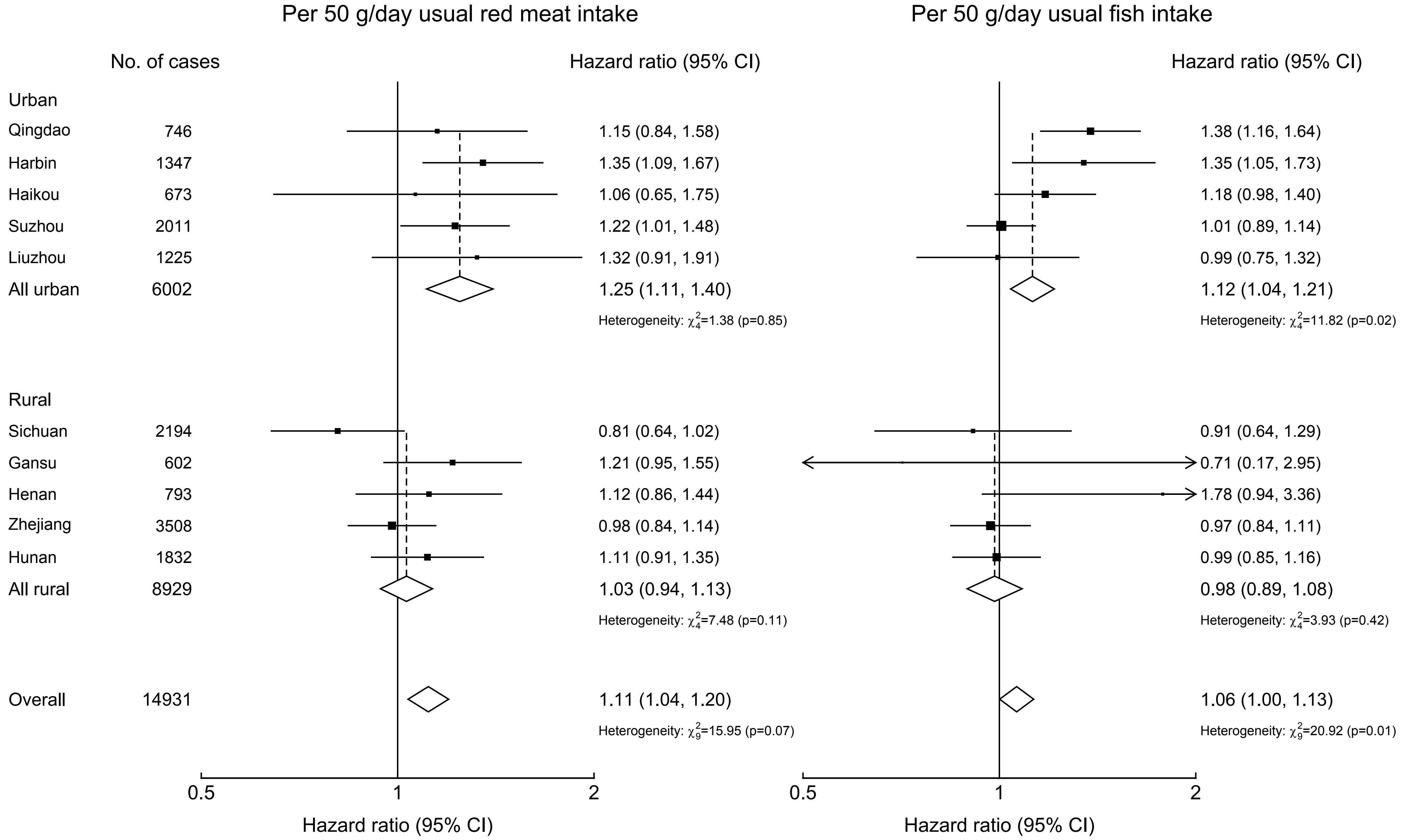

Analyses were stratified by age-at-risk, sex, and region, where appropriate, and adjusted for education, income, smoking, alcohol, physical activity, family history of diabetes, BMI, consumption of fresh fruit, poultry, and, where appropriate, red meat and fish.
Black boxes represent the HRs, with the size inversely proportional to the variance of the log HRs and the vertical lines represent the 95% confidence intervals (CIs).

Members of the China Kadoorie Biobank Collaborative Group

International Steering Committee: Junshi Chen, MD, Zhengming Chen, DPhil (principal investigator), Robert Clarke, FRCP, Rory Collins, FRS, Yu Guo, MSc, Liming Li, PhD (principal investigator), Jun Lv, PhD, Richard Peto, FRS, and Robin Walters, PhD;

International Coordinating Centre, Oxford, England: Daniel Avery, MSc, Derrick Bennett, PhD, Ruth Boxall, MD, Yumei Chang, PhD, Yiping Chen, DPhil, Zhengming Chen, DPhil, Robert Clarke, FRCP, Huaidong Du, PhD, Simon Gilbert, MSc, Alex Hacker, BA, Michael Holmes, PhD, Christiana Kartsonaki, DPhil, Rene Kerosi, MSc, Ling Kong, BSc, Garry Lancaster, BA, John McDonnell, GD(Comp SC), Iona Millwood, DPhil, Qunhua Nie, BA, Jayakrishnan Radhakrishnan, MSc, Paul Ryder, BA, Sam Sansome, BSc, Dan Schmidt, MSc, Rajani Sohoni, BSc, Iain Turnbull, MBBS, Robin Walters, PhD, Jenny Wang, MSc, Lin Wang, MSc, Neil Wright, MSc, Ling Yang, PhD, and Xiaoming Yang, PhD;

National Coordinating Centre, Beijing, China: Zheng Bian, MD, Yu Guo, MSc, Xiao Han, BA, Can Hou, BSc, Jun Lv, PhD, Pei Pei, BSc, Yunlong Tan, MD, and Canqing Yu, PhD;

10 Regional Coordinating Centres:

Qingdao: Qingdao Centre for Disease Control and Prevention (CDC): Zengchang Pang, Ruqin Gao, Shanpeng Li, Shaojie Wang, Yongmei Liu, Ranran Du, Yajing Zang, Liang Cheng, Xiaocao Tian, Hua Zhang, Yaoming Zhai, Feng Ning, Xiaohui Sun, Feifei Li. Licang CDC: Silu Lv, Junzheng Wang, and Wei Hou;

Harbin: Heilongjiang Provincial CDC: Mingyuan Zeng, Ge Jiang, Xue Zhou. Nangang CDC: Liqiu Yang, Hui He, Bo Yu, Yanjie Li, Qinai Xu, Quan Kang, and Ziyan Guo;

Haikou: Hainan Provincial CDC: Dan Wang, Ximin Hu, Hongmei Wang, Jinyan Chen, Yan Fu, Zhenwang Fu, and Xiaohuan Wang; Meilan CDC: Min Weng, Zhendong Guo, Shukuan Wu, Yilei Li, Huimei Li, and Zhifang Fu;

Suzhou: Jiangsu Provincial CDC: Ming Wu, Yonglin Zhou, Jinyi Zhou, Ran Tao, Jie Yang, and Jian Su; Suzhou CDC: Fang Liu, Jun Zhang, Yihe Hu, Yan Lu, Liangcai Ma, Aiyu Tang, Shuo Zhang, Jianrong Jin, and Jingchao Liu;

Liuzhou: Guangxi Provincial CDC: Zhenzhu Tang, Naying Chen, and Ying Huang; Liuzhou CDC: Mingqiang Li, Jinhuai Meng, Rong Pan, Qilian Jiang, Jian Lan, Yun Liu, Liuping Wei, Liyuan Zhou, Ningyu Chen, Ping Wang, Fanwen Meng, Yulu Qin, and Sisi Wang;

Sichuan: Sichuan Provincial CDC: Xianping Wu, Ningmei Zhang, Xiaofang Chen, and Weiwei Zhou. Pengzhou CDC: Guojin Luo, Jianguo Li, Xiaofang Chen, Xunfu Zhong, Jiaqiu Liu, and Qiang Sun;

Gansu: Gansu Provincial CDC: Pengfei Ge, Xiaolan Ren, and Caixia Dong; Maiji CDC: Hui Zhang, Enke Mao, Xiaoping Wang, Tao Wang, and Xi Zhang;
Henan: Henan Provincial CDC: Ding Zhang, Gang Zhou, Shixian Feng, Liang Chang, and Lei Fan; Huixian CDC: Yulian Gao, Tianyou He, Huarong Sun, Pan He, Chen Hu, Xukui Zhang, Huifang Wu, and Pan He;
Zhejiang: Zhejiang Provincial CDC: Min Yu, Ruying Hu, and Hao Wang; Tongxiang CDC: Yijian Qian, Chunmei Wang, Kaixu Xie, Lingli Chen, Yidan Zhang, Dongxia Pan, and Qijun Gu;
Hunan: Hunan Provincial CDC: Yuelong Huang, Biyun Chen, Li Yin, Huilin Liu, Zhongxi Fu, and Qiaohua Xu; Liuyang CDC: Xin Xu, Hao Zhang, Huajun Long, Xianzhi Li, Libo Zhang, and Zhe Qiu.
